# Dataset of near-infrared (NIR) spectral data for prediction of organic matter and total carbon in agricultural soil using homemade NIR spectrometer

**DOI:** 10.1016/j.dib.2025.111840

**Published:** 2025-06-25

**Authors:** Natchanon Santasup, Parichat Theanjumpol, Choochad Santasup, Sila Kittiwachana, Nipon Mawan, Nuttapon Khongdee

**Affiliations:** a Department of Plant and Soil Science, Faculty of Agriculture, Chiang Mai University, Chiang Mai, 50200, Thailand; b Postharvest Technology Research Center, Faculty of Agriculture, Chiang Mai University, Chiang Mai, 50200, Thailand; c Department of Chemistry, Faculty of Science, Chiang Mai University, Chiang Mai, 50200, Thailand; d Department of Highland Agriculture and Natural Resources, Faculty of Agriculture, Chiang Mai University, Chiang Mai, 50200, Thailand

**Keywords:** Chemometric, Pre-processing technique, Model development, Soil spectroscopy, Soil fertility

## Abstract

The paper presents the spectroscopic data obtained from a homemade NIR spectrometer developed for agricultural quality analysis, along with the calibration and validation of a model database for predicting agricultural soil properties. We collected NIR spectral data from 190 soil samples taken at a depth of 0-20 cm from agricultural areas in northern Thailand, including vegetable farms, orchards, and field crops. The acquisition process started by air-drying the soil and sieving it through 2.0 mm and 0.5 mm mesh. Six preprocessing techniques, including Savitzky-Golay smoothing, multiplicative scatter correction (MSC), standard normal variate (SNV), first derivative, second derivative, and mean centering, were used with partial least squares (PLS) regression to create the prediction model for soil organic matter and total carbon. Seventy percent of the sample was divided into calibration and the remaining thirty percent was validation. The most suitable model for assessing soil organic matter (SOM) and total carbon is Savitzky-Golay smoothing through the PLSR model, with a coefficient of determination (R^2^) of 0.79 and 0.78, a root mean square error (RMSE) of 0.701% and 0.382% for validation samples, respectively. Thus, the NIR dataset spanning 900-1,700 nm proved to be an ideal wavelength range for developing a portable/handheld NIR spectrometer, with potential for further accuracy improvements through model refinement.

Specifications Table

**Table:** 

Subject	Soil Science
Specific subject area	Soil Spectroscopy, Chemometric, Machine Learning
Type of data	Raw Analysed Presented as *.xlsx* and .*unsc* file formats
Data collection	Spectral data were collected from 190 agricultural topsoil samples (0-20 cm depth) in northern Thailand using a homemade NIR spectrometer equipped with a single-element NIR detector (DLP NIRscan Nano). Samples were dried and scanned in a 400 mL beaker across a wavelength range of 900-1,700 nm. The dataset was split into calibration (70%) and validation (30%) sets. Predictive models for soil organic matter and total carbon content were developed using partial least squares regression combined with six data pre-processing methods.
Data source location	Country: Thailand
Data accessibility	Repository name: Mendeley Data Data identification number: 10.17632/yt78nwnhbd.1 Direct URL to data: https://data.mendeley.com/datasets/yt78nwnhbd/1
Related research article	None

## 1 Value of the Data

• This study demonstrates the effectiveness of utilizing a wavelength range of 900-1,700 nm from a homemade NIR spectrometer to predict soil properties, and it could be a guideline for other researchers or engineers to develop a NIR spectrometer capable of using in-hole chains for agricultural production.
• Utilizing NIR spectroscopy to predict soil properties necessitated a comprehensive and diverse dataset of soil samples to develop a predictive model. However, the processes of soil sampling and laboratory analysis are laborious. If researchers exchange their soil samples and data, it would reduce the time required for research and yield more comprehensive prediction models covering a wider range of soil types.
• Researchers can improve model accuracy by applying advanced preprocessing methods and both linear and nonlinear modeling techniques to this NIR spectral data and wet chemistry results.

## 2 Background

Precision agriculture requires understanding soil parameters to effectively manage soil conditions, plant nutrients, and water resources for crop production [1,2]. Soil property analysis is generally performed in laboratories, yielding uniform and precise data. Laboratory analysis has certain limitations: (1) it is time-intensive, (2) requires specialist equipment, and (3) it uses substantial amounts of chemicals [3,4]. Presently, Visible and Near-Infrared Reflectance Spectroscopy (VIS-NIR) is a commonly utilized method for assessing soil characteristics [5] and the quality of agricultural products [6]. This method is rapid, efficient, non-destructive, and environmentally sustainable method for soil analysis [7,8]. VIS-NIR spectroscopy can be classified by wavelength into two ranges: (1) VIS-NIR (400–2,500 nm) and (2) NIR (800–2,500 nm), both applicable for soil quality assessment. However, commercial NIR spectrometers are typically expensive [9]. In response, portable/handheld NIR spectrophotometers have been developed to reduce cost, rendering them appropriate for soil property and agricultural product quality analysis. A portable NIR spectrophotometer capable of assessing both soil parameters and agricultural product quality will significantly improve field management efficiency from production to harvest. This study aims to evaluate the efficacy of homemade NIR spectrophotometer, first developed for assessing the quality of agricultural products, in estimating organic matter and total carbon in agricultural soils.

## 3 Data Description

**Fig. 1:**
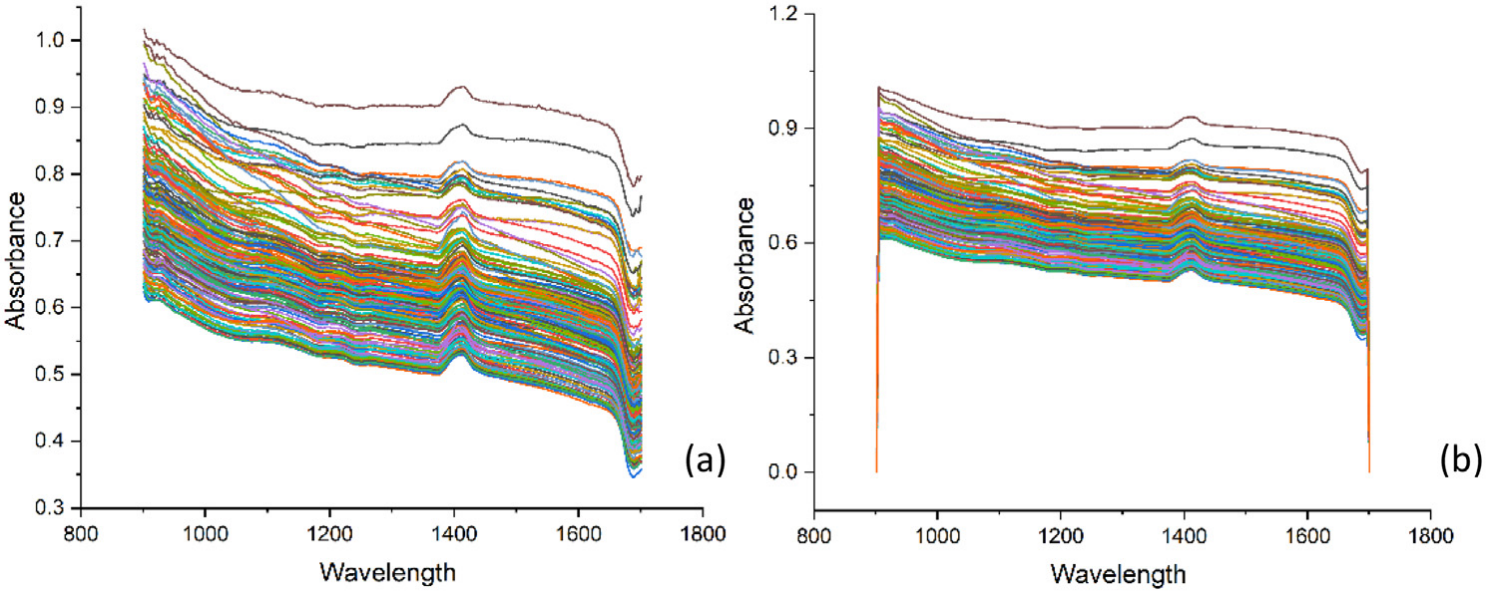
Raw NIR spectra of soil sample (a), spectral after smoothing pre-processing (b).

A near-infrared spectral dataset of soil samples was obtained as an absorbance spectrum in the wavelength range of 900 to 1,700 nm (Fig. 1). The absorbed wavelengths correspond to the vibrations of chemical bonds, specifically C−H, N−H, and O−H bonds, which are the functional group in organic compounds. When NIR light interacts with a sample, a portion of the light is absorbed, while the residual light is either reflected or transmitted. Analyzing the pattern of absorbed and reflected light yields information regarding the composition and properties of the sample [10]. Nevertheless, raw spectra often contain noise from differences in sample particle size, moisture content, and instrument drift. that might mask the authentic spectral information pertinent to soil characteristics. These variations can arise pre-processing approaches mitigate undesirable effects and augment spectral relevant properties. Suitable pre-processing techniques enhance the signal-to-noise ratio, producing more precise and resilient calibration models for forecasting soil parameters [11].

The soil spectral dataset of 190 soil samples is provided in 2 files:

1) The file “Homemade NIR Spectral.xlsx” includes 7 spectral and average scans of soil samples from the Homemade NIR spectrometer
2) The file “Data research.xlsx” provides the average of raw and pre-processing spectra combined with the chemical analysis used to build the model.

The PLSR models in this study are provided in “.Unsc” (Table 1) This format is accessible in the Unscrambled program.

**Table 1:** Description and PLSR model file name for SOM and TC prediction in soil.

File description	File name
PLSR model for SOM from Raw spectral	Raw-SOM-Model.41M
PLSR model for SOM from smoothing preprocess	Smoothing-SOM-Model.41M
PLSR model for SOM from MSC preprocess	MSC-SOM-Model.41M
PLSR model for SOM from SNV preprocess	SNV-SOM-Model.41M
PLSR model for SOM from Mean center preprocess	Mean center-SOM-Model.41M
PLSR model for SOM from 1^st^ Derivative preprocess	1-Derivative-SOM-Model.41M
PLSR model for SOM from 2^nd^ Derivative preprocess	2-Derivative-SOM-Model.41M
PLSR model for TC from Raw spectral	Raw-TC-Model.41M
PLSR model for TC from smoothing preprocess	Smoothing-TC-Model.41M
PLSR model for SOM from MSC preprocess	MSC-TC-Model.41M
PLSR model for SOM from SNV preprocess	SNV-TC-Model.41M
PLSR model for SOM from Mean center preprocess	Mean center-TC-Model.41M
PLSR model for SOM from 1^st^ Derivative preprocess	1-Derivative-TC-Model.41M
PLSR model for SOM from 2^nd^ Derivative preprocess	2-Derivative-TC-Model.41M

## 4 Experimental Design, Materials and Methods

### 4.1 Instrument setup

This study utilized a custom-built NIR spectrometer (homemade-NIR) with a single-element NIR detector (DLP NIRscan Nano). This detector measured reflected near-infrared light within the 900-1,700 nm wavelength range (including short-wave and long-wave NIR), the design of the spectrometer, both internal and external, the body of the spectrometer was made from stainless steel. It has an LED touchscreen, controlled by a custom program developed on the Raspberry Pi platform using Python, which facilitates operation and a USB connection enables data transfer to a personal computer for advanced analysis. This homemade NIR spectrometer was developed by the Post-Harvest Technology Research Center, Faculty of Agriculture, Chiang Mai University, to evaluate the quality of agricultural products, particularly grain products such as rice, coffee, soybean, and mung bean [12,13]

### 4.2 Soil sample collection and chemical analysis

Topsoil samples (0-20 cm) were collected from 190 agricultural plots in northern Thailand, including vegetable farms, orchards, and field crops. The samples were prepared for chemical analysis by air-drying and sieving through a 2.0 mm. to remove plant debris, gravel, rocks, and other coarse materials. The <2.0 mm fraction was used for general soil property analyses, including pH, electrical conductivity (EC), and available phosphorus (P), etc. For soil organic matter (SOM) and total carbon (TC) determination, a subsample was further sieved through a 0.5 mm mesh to ensure homogeneity. Soil organic matter (SOM) was analyzed based on the soil organic carbon (SOC) content measured via the Walkley and Black chromic acid wet oxidation method [14]. This involved oxidizing organic carbon in soil with a potassium dichromate (K_2_Cr_2_O_7_) solution in concentrated sulfuric acid. The remaining unreduced dichromate was measured by back-titrating with ferrous sulfate, using the o-phenanthroline-ferrous complex as an indicator. The resulting SOC values were converted to SOM using the conventional van Bemmelen factor of 1.724, which assumes that SOM contains 58% carbon [15]. The total carbon percentage was evaluated by putting 0.05 g of soil samples into a C-free boat and placing them in a C−S Analyzer (analytikjena). Table 2 displays the typical amounts of organic matter and total carbon found in the soil samples analyzed for this study.

**Table 2:** Range of soil organic matter and total carbon in agricultural soils.

Soil Chemical Constituent	number	Average	S.D.*
Soil organic matter (%)	190	2.40	1.61
Total carbon (%)	190	1.62	0.84

⁎ Noted: S.D. = Standard Deviation

### 4.3 Spectral data acquisition

**Fig. 2:**
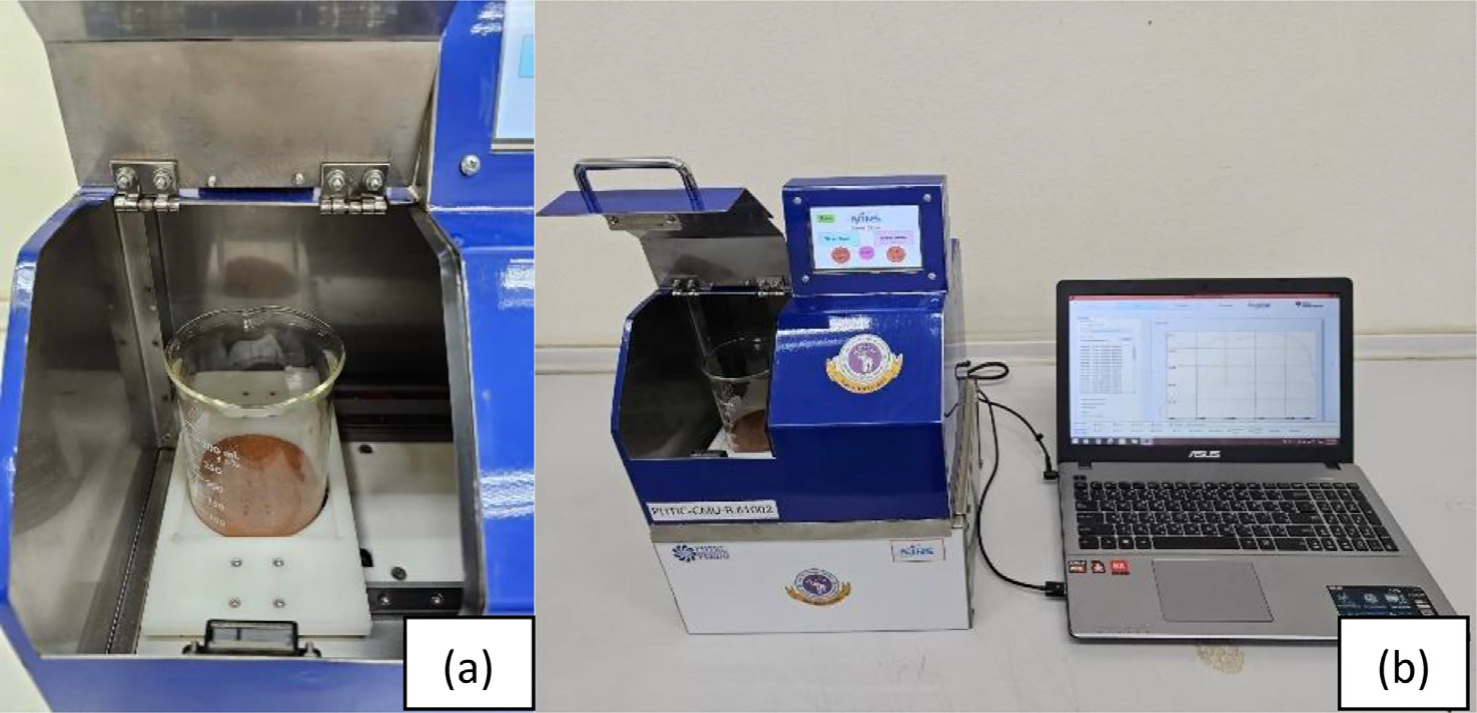
Sample holder in the homemade NIR spectrometer (a), and soil Spectral data acquisition (b).

The soil sample (particle size < 0.5 mm) was thereafter transferred to a 400 mL beaker (A commercial Quartz beaker PYREX) for measuring NIR spectra in wavelengths of 900-1700 nm, using a homemade NIR spectrometer (Fig. 2). This homemade NIR system was based on detecting using a low-cost micro-electronic mechanism system (MEMS) sensor. The NIR spectra were recorded with 7 replicates to provide the average spectrum of each sample.

### 4.4 Calibration model

Before model development, six preprocessing techniques were employed to enhance the precision and robustness of predictive outcomes. These techniques included Savitzky-Golay smoothing, multiplicative scatter correction (MSC), standard normal variate (SNV), first derivative, second derivative and mean centering. Partial Least Squares (PLS) regression was utilized to construct predictive models for soil organic matter and total carbon content. The dataset was divided into calibration and validation sets of 70% and 30% of the total soil samples, respectively. The efficacy of the predictive models was assessed utilizing the coefficient of determination (R²) and the root mean square error (RMSE). The ideal prediction model was chosen based on the maximum R² and minimum RMSE values.

Partial Least Squares Regression (PLSR) is a statistical technique that integrates principal component analysis (PCA) aspects with multiple regression. It is especially beneficial when managing high-dimensional data when the quantity of predictor variables significantly exceeds the number of observations. PLSR seeks to identify latent variables that maximize the covariance between predictor variables and the response variable. The latent variables are subsequently utilized to construct a regression model [16].

### 4.5 Model evaluation

**Fig 3:**
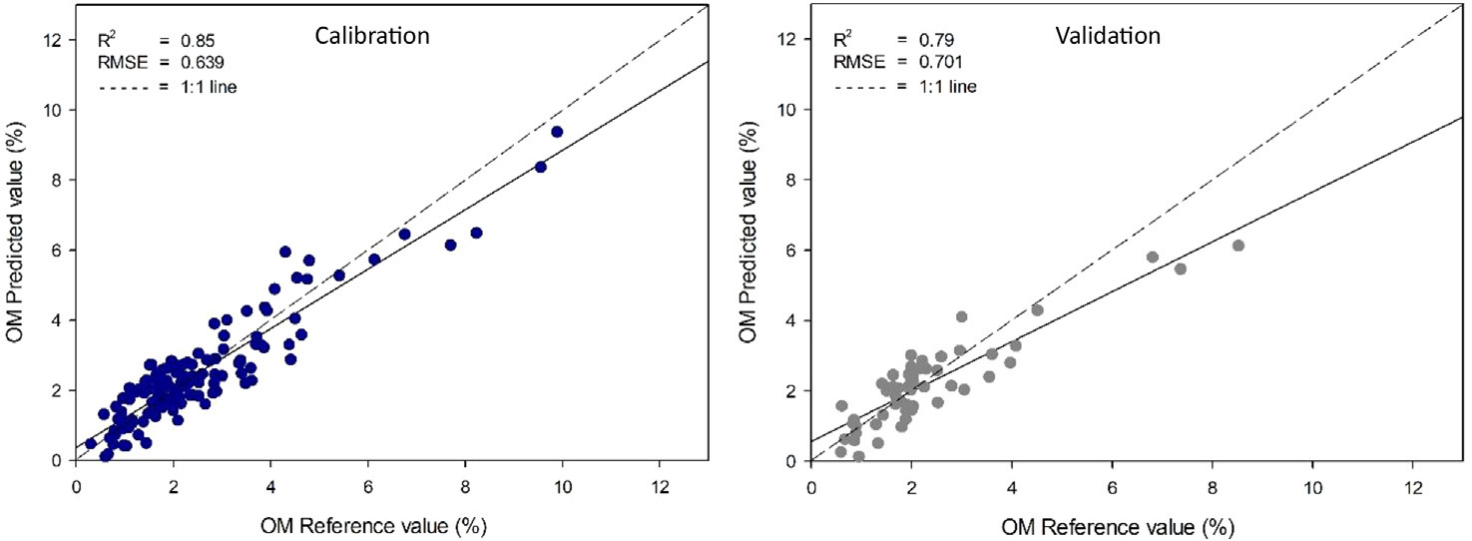
Comparisons of soil organic matter were assessed through wet oxidation and estimated using a homemade NIR spectrometer employing a smoothing preprocessing technique with a PLS model.

**Fig 4:**
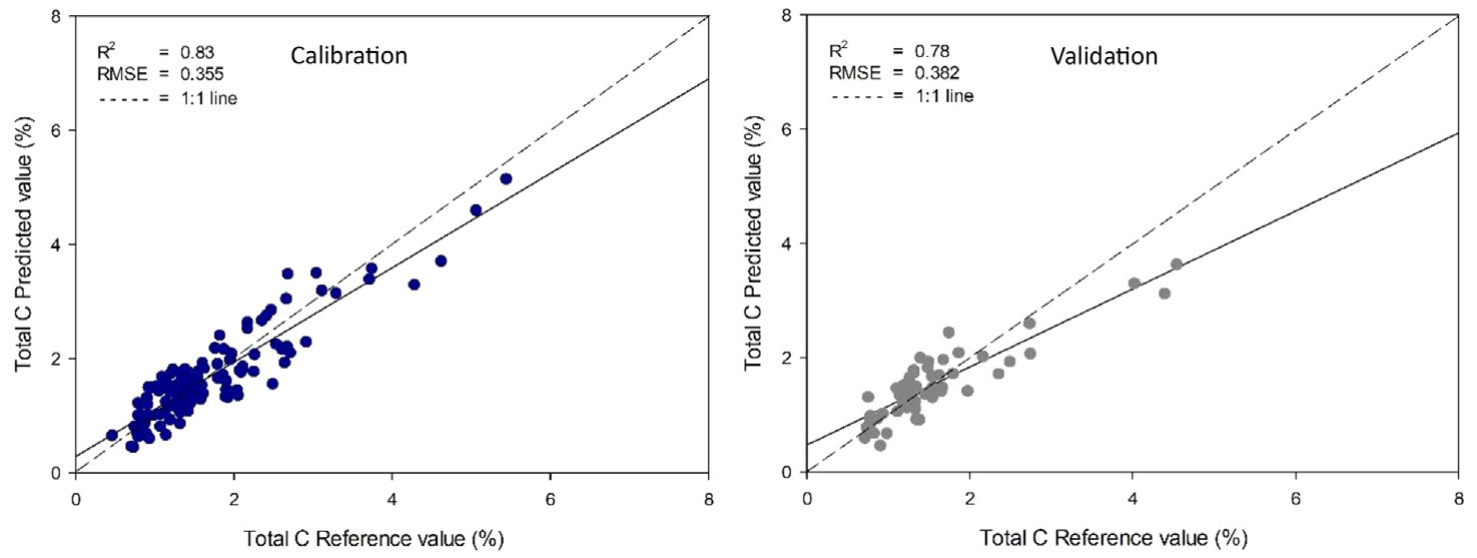
Comparisons of total carbon were assessed through wet oxidation and estimated using a homemade NIR spectrometer employing a smoothing preprocessing technique with a PLS model.

Table 3 presents the calibration and validation statistics of PLS prediction models. These models were created to correlate the absorbance from near-infrared spectroscopy with the reference values of organic matter and total carbon in soil derived from established analytical techniques. The optimal model for organic matter was derived by smoothing pre-processing using PLS, achieving the highest R^2^ (0.79) and the lowest RMSE (0.701%) for validation samples (Fig. 3). For total carbon, the integration of smoothing pre-processing with PLSR resulted in the most precise model (Table 4), with the highest R^2^ of 0.78 and the lowest RMSE of 0.382% among validation samples. The most precise model, developed through pre-processing smoothing using PLS regression, is depicted in Fig. 4. The results of this study underscore the versatility of a home-made near-infrared (NIR) spectrometer, demonstrating its capability to analyze diverse sample types, including agricultural products and soil properties such as organic matter and total carbon content.

**Table 3:** The key model parameters and statistical performance metrics for predicting soil organic matter (SOM) using partial least squares (PLS) regression with various data preprocessing techniques.

Pre-processing	Calibration				Validation				Factor
	Correlation	R^2^	RMSEC	SEC	Correlation	R^2^	RMSEP	SEP	
Raw data	0.93	0.86	0.618	0.621	0.89	0.78	0.712	0.714	9
Smoothing	0.92	0.85	0.640	0.642	0.89	0.79	0.701	0.701	10
MSC	0.85	0.72	0.867	0.871	0.81	0.64	0.914	0.914	7
SNV	0.88	0.78	0.775	0.778	0.80	0.63	0.918	0.918	9
1 ^st^ derivative	0.88	0.77	0.791	0.794	0.86	0.72	0.817	0.819	6
2 ^nd^ derivative	0.91	0.83	0.682	0.684	0.78	0.60	0.965	0.961	7
Mean centering	0.93	0.86	0.618	0.621	0.89	0.78	0.712	0.715	9

**Table 4:** The key model parameters and statistical performance metrics for predicting total carbon using partial least squares (PLS) regression with various data preprocessing techniques.

Pre-processing	Calibration				Validation				Factor
	Correlation	R^2^	RMSEC	SEC	Correlation	R^2^	RMSEP	SEP	
Raw data	0.92	0.84	0.339	0.340	0.87	0.75	0.401	0.404	9
Smoothing	0.91	0.83	0.355	0.356	0.89	0.78	0.382	0.385	10
MSC	0.84	0.71	0.458	0.460	0.78	0.61	0.504	0.507	7
SNV	0.88	0.77	0.406	0.408	0.77	0.60	0.511	0.514	9
1 ^st^ derivative	0.87	0.75	0.426	0.427	0.82	0.66	0.469	0.472	6
2 ^nd^ derivative	0.91	0.82	0.363	0.365	0.72	0.52	0.558	0.560	7

Savitzky-Golay smoothing is commonly used for near-infrared spectroscopy of soil samples because it efficiently eliminates local signal noise from multiple sources, including instrument variability and sample heterogeneity by applying a polynomial to a moving window of data points through the least squares approach. Moreover, it can maintain critical spectral characteristics, including absorption bands associated with soil organic matter, moisture content, and other essential aspects. The polynomial degree and the sliding window size are critical parameters that regulate the extent of smoothing [17].

## Limitations

• This study employed a specific wavelength selection and utilized a homemade NIR spectrometer, which may require advanced preprocessing techniques and model creation to enhance the accuracy of the predictive model.
• The spectral library currently represents only agricultural soils in northern Thailand. To develop it on a global scale, the number of samples must be increased, and models must be further developed to evaluate additional soil properties in the future.
• The library was built using air-dried samples, which makes it unsuitable for assessing soil conditions directly in the field.

## Ethics Statement

This study does not involve human subjects, animal experiments, or data collected from social media platforms.

## Credit Author Statement

**Natchanon Santasup:** Data collecting, Writing- Original draft preparation. **Parichat Theanjumpol** Conceptualization, Supervision **Choochad Santasup:** Conceptualization. **Sila Kittiwachana**: Supervision. **Nipon Mawan**: Methodology. **Nuttapon Khongdee**: Conceptualization, Writing- Reviewing and Editing.

## Data Availability

Mendeley DataDataset of near-infrared (NIR) spectral data for prediction of organic matter and total carbon in agricultural soil using homemade NIR spectrometer (Original data).

## References

[1] Padhiary M., Saha D., Kuma R., Sethi L.N., Kumar A. (2024). Enhancing precision agriculture: a comprehensive review of machine learning and AI vision applications in all-terrain vehicle for farm automation. Smart Agric. Technol..

[2] Albuquerque J.R.D.P., Makara C.N., Ferreira V.G., Brazaca L.C., Carrilho E. (2024). Low-cost precision agriculture for sustainable farming using paper-based analytical devices. RSC. Adv..

[3] Soriano-Disla J.M., Janik L.J., Viscarra Rossel R.A., Macdonald L.M., McLaughlin M.J. (2014). The performance of visible, near-, and mid-infrared reflectance spectroscopy for prediction of soil physical, chemical, and biological properties. Appl. Spectrosc. Rev..

[4] Rossel R.A.V., Walvoort D.J.J., McBratney A.B., Janik L.J., Skjemstad J.O. (2006). Visible, near infrared, mid infrared or combined diffuse reflectance spectroscopy for simultaneous assessment of various soil properties. Geoderma.

[5] Yu B., Yan C., Yuan J., Ding N., Chen Z. (2023). Prediction of soil properties based on characteristic wavelengths with optimal spectral resolution by using Vis-NIR spectroscopy. Spectrochimica Acta Part A.

[6] Czaja T.P., Engelsen S.B. (2025). Why nothing beats NIRS technology: The green analytical choice for the future sustainable food production. Spectrochimica Acta Part A.

[7] Wang Z., Chen S., Lu R., Zhang X., Ma Y., Shi Z. (2024). Non-linear memory-based learning for predicting soil properties using a regional vis-NIR spectral library. Geoderma.

[8] Zhu J., Jin Y., Zhu W., Lee D.K. (2024). VIS-NIR spectroscopy and environmental factors coupled with PLSR models to predict soil organic carbon and nitrogen. Int. Soil Water Conserv. Res..

[9] Bertinetto C.G., Schoot M., Dingemans M., Meeuwsen W., Buydens L.M., Jansen J.J. (2022). Influence of measurement procedure on the use of a handheld NIR spectrophotometer. Food Res. Int..

[10] Rossel R.A.V., Behrens T. (2010). Using data mining to model and interpret soil diffuse reflectance spectra. Geoderma.

[11] Rinnan A., van den Berg F., Engelsen S.B. (2009). Review of the most common pre-processing techniques for near-infrared spectra. Trends Analytical Chem..

[12] Phuangsaijai N., Theanjumpol P., Muenmanee N., Kittiwachana S. (2021). Fabrication of a low-cost NIR spectrometer for detection of agricultural product quality. Chiang Mai J. Sci..

[13] Kaewpangchan P., Phuangsaijai N., Seehanam P., Theanjumpol P., Maniwara P., Kittiwachana S. (2021). Screening of coffee impurity using a homemade NIR sensor system. Chiang Mai J. Sci..

[14] Walkley A., Black I.A. (1934). An examination of the degtjareff method for determining soil organic matter and a proposed modification of the chromic acid titration method. Soil. Sci..

[15] Van Bemmelen J.M. (1890). Über die Bestimmung des Wassers, des Humus, des Schwefels, der in den colloïdalen Silikaten gebundenen Kieselsäure, des Mangans u.s. w. im Ackerboden. Die Landwirthschaftlichen Versuchs-Stationen.

[16] Abdi H. (2010). Partial least squares regression and projection on latent structure regression (PLS Regression). Wiley Interdiscip. Rev..

[17] Schmid M., Rath D., Diebold U. (2022). Why and how savitzky-golay filters should be replaced. ACS. Meas. Sci. Au.

